# Data Management Rubric for Video Data in Organismal Biology

**DOI:** 10.1093/icb/icx060

**Published:** 2017-07-13

**Authors:** Elizabeth L. Brainerd, Richard W. Blob, Tyson L. Hedrick, Andrew T. Creamer, Ulrike K. Müller

**Affiliations:** * Department of Ecology and Evolutionary Biology, Brown University, Providence, RI 02912, USA; † Department of Biological Sciences, Clemson University, Clemson, SC 29634, USA; ‡ Department of Biology, University of North Carolina at Chapel Hill, Chapel Hill, NC 27599, USA; § Brown University Library, Brown University, Providence, RI 02912, USA; ¶ Department of Biology, California State University Fresno, 2555 E San Ramon Avenue, Fresno, CA 93740, USA

## Abstract

Standards-based data management facilitates data preservation, discoverability, and access for effective data reuse within research groups and across communities of researchers. Data sharing requires community consensus on standards for data management, such as storage and formats for digital data preservation, metadata (i.e., contextual data about the data) that should be recorded and stored, and data access. Video imaging is a valuable tool for measuring time-varying phenotypes in organismal biology, with particular application for research in functional morphology, comparative biomechanics, and animal behavior. The raw data are the videos, but videos alone are not sufficient for scientific analysis. Nearly endless videos of animals can be found on YouTube and elsewhere on the web, but these videos have little value for scientific analysis because essential metadata such as true frame rate, spatial calibration, genus and species, weight, age, etc. of organisms, are generally unknown. We have embarked on a project to build community consensus on video data management and metadata standards for organismal biology research. We collected input from colleagues at early stages, organized an open workshop, “Establishing Standards for Video Data Management,” at the Society for Integrative and Comparative Biology meeting in January 2017, and then collected two more rounds of input on revised versions of the standards. The result we present here is a rubric consisting of nine standards for video data management, with three levels within each standard: good, better, and best practices. The nine standards are: (1) data storage; (2) video file formats; (3) metadata linkage; (4) video data and metadata access; (5) contact information and acceptable use; (6) camera settings; (7) organism(s); (8) recording conditions; and (9) subject matter/topic. The first four standards address data preservation and interoperability for sharing, whereas standards 5–9 establish minimum metadata standards for organismal biology video, and suggest additional metadata that may be useful for some studies. This rubric was developed with substantial input from researchers and students, but still should be viewed as a living document that should be further refined and updated as technology and research practices change. The audience for these standards includes researchers, journals, and granting agencies, and also the developers and curators of databases that may contribute to video data sharing efforts. We offer this project as an example of building community consensus for data management, preservation, and sharing standards, which may be useful for future efforts by the organismal biology research community.

## Introduction

Organismal biology embraces complexity, and increasingly we are challenged with the problem of managing large and complex datasets. Successful data management can facilitate collaboration and data sharing, and thereby promote integration and synthesis (Strasser 2015). To promote data sharing, research communities benefit from developing frameworks of consensus standards for data management to ensure that data are findable, accessible, interoperable, and reusable (Wilkinson et al. 2016). These include standards for data acquisition, digital data preservation, the kinds of metadata (i.e., contextual data about the data) that should be recorded and stored along with the primary data, data and metadata curation, and data access (Reichman et al. 2011; Michener and Jones 2012; Yarmey and Baker 2013; Riley 2017). Without such standards, it is difficult to achieve interoperability—the exchange and use of information across systems. A lack of interoperability can have far reaching impacts, limiting the coordination of effort across computer systems, institutions, and research teams.

Data sharing through online databases is an increasingly common practice in ecology, evolution, and bioinformatics, resulting in large-scale and high-impact results (e.g., Qin et al. 2010; Hampton et al. 2013; Hudson et al. 2014; Hampton et al. 2015). Like organismal biology, ecology and environmental sciences are “long-tail” research disciplines generating a long tail of what Heidorn calls “dark data” (Heidorn 2008): projects with single or few Principal Investigators (PIs) on relatively small, short-term research budgets that generate a lot of data that are difficult to access outside the PI’s research lab. Combining data from a wide range of small projects comes with its own challenges: data dispersal, data heterogeneity, and data provenance (Reichman et al. 2011).

To address these issues, ecologists have developed effective standards for data sharing (e.g., Reichman et al. 2011; Michener and Jones 2012; White et al. 2013). There are extensive studies on adoption barriers (technological barriers: Bach et al. 2012; attitude barriers: Sayogo and Pardo 2012; Tenopir et al. 2015), best practices to develop shared standards (Wolkovich et al. 2012; White et al. 2013; Yarmey and Baker 2013), required IT infrastructure (Bach et al. 2012), policy (Sayogo and Pardo 2012; Roche et al. 2014; Tenopir et al. 2015), and institutional and personnel support (Specht et al. 2015). The main messages from the ecology and environmental science communities concerning data sharing are that it takes several years to develop effective shared standards for data archiving (Yarmey and Baker 2013) and data completeness and reusability (Roche et al. 2015), and that data sharing is significantly more common in disciplines with good data sharing practices and policies (Tenopir et al. 2015).

Several initiatives have developed best practices for research data management or metadata standards, such as Darwin Core (rs.tdwg.org/dwc), the Digital Curation Center (dcc.ac.uk), and BioSharing (biosharing.org). Most relevant to organismal biology, the digital morphology research community has recently published community standards and best practices for three-dimensional digital data publication, storage, and reuse (Davies et al. 2017), and natural history museums have developed the Integrated Digitized Biocollections (iDigBio.org) consortium to foster integration and interconnectivity of digital specimen records, images, and associated data (Page et al. 2015).

In addition, recent funder policies and initiatives have emerged to support the management and sharing of data, metadata, and software collected or created by sponsored researchers, in addition to the sharing of their publications. Examples in the United States include the Public Access Policies developed by federal granting agencies over the last 2 years in response to an Obama White House Policy Memorandum from its Office of Science and Technology Policy (OSTP) (obamawhitehouse.archives.gov/blog/2013/02/22/expanding-public-access-results-federally-funded- research), and initiatives such as the National Institutes of Health’s (NIH) Big Data to Knowledge (BD2K) program (datascience.nih.gov/bd2k), as well as the National Science Foundation’s (NSF) integration of disseminating and sharing of research data into its proposal and award policies and procedures (nsf.gov/bfa/dias/policy/dmp.jsp). However, such “open data” practices have not yet been widely implemented in organismal biology.

Organismal biology increasingly relies on video recordings as a means to collect raw data to address research questions in many areas such as functional morphology, comparative biomechanics, and animal behavior. A central goal of organismal biology is to quantify complex phenotypes (Halanych and Goertzen 2009; Schwenk et al. 2009; Mykles et al. 2010; Padilla et al. 2014). For aspects of phenotype that have a time-dependent component, video imaging is the best capture medium. Video can capture a range of time-varying phenotypes, from relatively simple motions, such as a frog jump, to complex social behaviors of animals. Video with synchronized audio is particularly important for animal behavior research. In some cases, several high-speed, high-resolution video cameras running at 1000 or more frames per second are used to capture rapid 3D (three-dimensional) motion. Over the last few decades, video technology has become widespread due to technological advances in high-speed, infrared, and x-ray videography, in machine vision, image analysis and motion analysis, and in storage and management (Hedrick 2008; Brainerd et al. 2010; Schwenk and Wagner 2010; Lauder 2015; Jackson et al. 2016; Knörlein et al. 2016). These advances have led to large collections of video data, which are potentially amenable to data mining and synthesis, especially as machine learning applications to video analysis become more established.

Individual video files from video cameras are large, often 2–10 gigabytes (GB) or more, and even a small study can routinely generate a terabyte (TB) or more of data. Furthermore, we may be on the cusp of an explosion in the volume of organismal video, given the advent of low-cost consumer video cameras with high frame rates, such as GoPro cameras (Jackson et al. 2016). Until recently, high-speed cameras cost several tens of thousands of dollars and were found only in high-end research labs. Now high-speed cameras are accessible to researchers and students at small colleges and even K-12 schools, broadening research opportunities in organismal biology, and exacerbating (or enhancing, depending on your perspective) the long tail of dark data (Heidorn 2008).

Although videos provide the primary data for many studies, videos alone are generally not sufficient for scientific analysis. Researchers also need some data about the data (metadata), such as frame rate, spatial scale, and potentially many other kinds of metadata. Vast amounts of animal video can be found on YouTube and elsewhere on the web, but these videos are often unsuitable for scientific work because we do not know the true frame rate or size scale, let alone the multi-camera calibration information necessary for 3D motion analysis. Moreover, for many publically available videos, data are also lacking on the video subjects, such as the exact species, the weight or length of the animal, its age, sex, etc. Some clever research has been done mining web video sources such as YouTube, in one case by using typical adult body sizes to estimate the spatial scale (Lucas et al. 2014), but all of the video data collected specifically for research would be far more valuable with appropriate metadata attached.

The goals of this ICB Perspectives article are (from broadest to most specific and back to broad): (1) to make the case for the value of building community consensus on data management and sharing standards in many areas of organismal biology; (2) to describe our process for building consensus on data management standards for video data; (3) to present the rubric and make suggestions for best practices and implementation; (4) to provide examples of the rubric applied to published works; and (5) to draw any general conclusions about building consensus, metadata standards, data citation, sharing, management, and preservation for organismal biology research that may emerge from our experience.

## Rationale and process for developing community standards for data management

We recognize three key goals that provide strong motivation for organismal biology researchers to develop standards for video data management.

1. To protect and ensure preservation of video data (preservation of both their integrity and access to them) generated for research in organismal biology.
2. To promote the documentation, sharing, reuse, and citation of video data for acceleration of research in organismal biology.
3. To ensure that the research community has input in shaping the standards to be used by institutions and granting agencies to evaluate the quality of data management plans and practices.

Our first two goals assume that researchers want to manage their data well, for the benefit of their own research groups as well as for the broader research enterprise. Thus, our focus is on establishing standards for practices that can help achieve this goal, rather than on ways to encourage investigators to implement those practices. Moreover, our focus concentrates on the video, metadata, and any auxiliary data collected synchronously with video (e.g., electromyograms, pressure, force) that researchers would intend to save and maintain through the course of conducting a study, rather than any additional video or files that would not have been saved as data were collected. We also write from the perspective of a “video first” research plan, where video is the primary data gathering mechanism. Research plans where video is an ancillary tool may be better served by adding it to established standards for management of the primary experimental data.

Our final goal recognizes increasing expectations for researchers to document the approaches they use to manage the datasets they generate. For example, funding agencies, such as the NSF, evaluate our data management efforts through annual and final reports, and statements of results of prior support in new submissions. If the organismal biology community does not participate in developing standards for data management practices, funding agencies will have to rely on a patchwork of reviewer and panelist opinions for evaluating our data management plans and their implementation. Such opinions may not be grounded in the needs or objectives of organismal biologists. In addition to funder expectations, there are also publisher requirements for the retention of data supporting publications (e.g., Joint Data Archiving Policy [datadryad.org/pages/jdap]) and for the citation, deposit, and long-term archiving of these data in public repositories (Nosek et al. 2015).

The current project to develop community standards for video data management arose from an initial workshop, “Data Management Plans for NSF Proposals,” organized by the NSF Division of Integrative Organismal Systems at the annual meeting of the Society for Integrative and Comparative Biology in January, 2016. At the workshop, the NSF staff encouraged participants to work toward community standards for data management. With that goal in mind, we offer the standards developed here both for their direct value in video data management, but also to document our process for building community consensus for such standards. Whether our process for building consensus has been successful will ultimately be determined by whether researchers in organismal biology actually use the standards, and whether the research community updates them in the future as required by changes in technology or research needs.

Following best practices for developing such standards, using participatory rather than hierarchical approaches (Yarmey and Baker 2013), we obtained input from stakeholders to develop standards for video data management through several rounds of feedback, akin to the process leading to the development of the Long-Term Ecological Research Ecological Metadata Language (LTER EML). Our first step was to create a simple list of video data management issues and types of metadata that might make the videos most useful for future research. In November 2016, we collected feedback on this preliminary list from colleagues and students at some of our local institutions, and from a few other colleagues we knew were interested in the project. Then we turned that list into a rubric consisting of management standards with three levels per standard (good, better, and best practices), with a Level 0 that reflects unacceptable practices (modeled on Table 1 in Nosek et al. 2015). The rubric became version 0.1 of the Video Data Management Standards.

**Table 1:** Rubric for best practices in video data management for organismal biology research

Standards	Level 0: unacceptable	Level 1: good	Level 2: better	Level 3: best
(1) Data storage	Single copy, local disk storage only (such as on a hard drive).	A local working copy plus an archival^a^ copy in professionally managed/cloud^b^ storage OR two additional local archival copies, one in a separate physical location. All plain disk copies migrated to fresh media on a set schedule. All server copies subjected to regular file integrity checks.	One archival^a^ copy in professionally managed/cloud^b^ storage plus at least two additional local copies in separate locations. All local copies migrated to fresh media on a planned schedule if on plain disks or subjected to regular file integrity checks if on a server.	Archival^a^ copy stored in a data repository^c^ with a stated mission of digital data preservation.
(2) Video file formats^d^	Video files compressed, resized, or at a different frame rate from the original video files (e.g., YouTube or Vimeo).	Original, archival^a^ video files, even if format includes codecs or file types that are not widely accessible by common viewing software.	Level 1 plus version converted to a widely accessible format with maximum data preservation in the conversion.	Level 2 plus compressed/converted version(s)^e^ for viewing and greater accessibility online.
(3) Metadata linkage	Metadata absent or separate from video files (such as in lab notebooks); substantial effort required to share.	Metadata contained in digital files in a widely used format. Metadata files linked to video files by similar file names OR by bundling each video file together with its metadata into an uncompressed archive, such as zip, tar or hdf5.	Same as Level 1 except metadata files linked to video files by similar file names AND by bundling each video file together with its metadata; OR metadata text embedded in the video file itself.	Metadata, including video file name, encoded in XML or other machine-readable format and contained within the video files themselves or by bundling each video file together with its metadata.
(4) Video data and metadata access	Not directly accessible online; substantial effort required to share.	Video data and metadata available in an Internet-accessible location, such as in commercial cloud^b^ storage or on a local drive on a network-connected computer.	Video data and metadata online in a public repository with a stated mission of providing public access to data^f^.	Level 2 plus metadata stored in a manner to make the videos discoverable on the web; i.e., metadata searchable and viewable without downloading a large video bundle^g^.
(5) Contact information and acceptable use	No contact information and no statement of terms of reuse.	Contact name and e-mail address and a clear statement about rights and acceptable reuse of the video.	Name, e-mail and assignment of an internationally-recognized content license^h^.	Level 2 plus ORCID ID for contact person and the assignment of a unique identifier such as a digital object identifier that can be used for the data’s discovery and citation.
(6) Camera settings	No metadata.	Frame rate (frames per second).	Frame rate and spatial calibration data and number of cameras and camera ID (camera used for this specific video) if part of multi-camera system.	Level 2 plus four or more of the following: video resolution (in pixels); shutter speed/exposure time; audio (Y/N); camera make and model; lens type; video type (e.g., monochrome, color, X-ray, PIV, infrared); file format; camera view (e.g., lateral); original video or post-processed; length (duration) of the video.
(7) Organism(s)	No metadata.	Genus and species (more than one binomial if more than one species in the video).	Genus, species, and at least one of the following: individual ID (multiple individual IDs if multiple individuals); some measure of size (e.g., length, weight).	Level 2 plus four or more of the following: sex; age; life stage; physical condition (e.g., prior invasive procedures, senescent, gravid, mutant); wild caught or captive bred; higher taxonomic groupings above genus; common name; links to publications using the same individual(s); accession number(s) if individual(s) were deposited in a museum.
(8) Recording conditions	No metadata.	Date OR location recorded (institution or field site or location name or GPS coordinates)^i^.	Date AND location recorded (institution or field site or location name or GPS coordinates)^i^. Name(s) of person or people who recorded the video recommended, but not required.	Level 2 plus two or more of the following: temperature; light regime; time of day; humidity; season; auxiliary data recorded (e.g., EMG, pressure, force; none); synchronization method for auxiliary data (if any); environment (e.g., water, land, air, tree canopy; treadmill; trackway; flume); recorded indoors or outdoors.
(9) Subject matter/ topic	No metadata.	Text description (abstract) of the contents of the video, including any related publication citations and information on the original purpose for which the video was collected. If the video belongs to a specific collection, experiment, field project, etc., include its name/title. Funding source and other acknowledgments should be included.	Level 1 plus 5–10 keywords. Suggestions for keywords include behavior (e.g., swimming, flying, displaying); experimental treatments applied (e.g., incline of treadmill, food type, denervated); entire organism visible or focus on part (e.g., head, knee, caudal fin).	Level 1 plus 5–10 keywords selected from an internationally recognized list (controlled vocabulary/taxonomy) of subjects/topics, (e.g., Encyclopedia of Life TraitBank).

a Archival copy is unmodified from original and remains unmodified.

b Commercial cloud services such as Google Drive, Dropbox, Amazon S3, Rackspace, EMC, MS OneDrive, or other storage managed by IT professionals, such as through an academic institution.

c Scientific repositories (e.g., Dryad, OSF, XMA/ZMAPortal) and university and library-based data repositories would meet this standard.

d Levels 1 and 2 are identical if original file formats are widely accessible and Levels 1–3 are identical if original files are also small enough for easy viewing and accessibility online.

e A warning should be provided if frame rate or pixel resolution has been changed for files that can be downloaded, since these affect spatial and temporal calibrations.

f Embargo periods are permitted; public access defined here as offered with a license that permits reuse of the data.

g Best is metadata and videos in a database with an interface designed to make them discoverable from outside the site and the metadata searchable and videos viewable from within the interface.

h Such as a Creative Commons CC0 or CC BY, or GNU General Public License or Open Data Commons ODC.

i Use of international standards is encouraged: ISO 8601 for date format; ISO 6709 for GPS coordinates; and ISO 27729 for institutions.

Then we organized a workshop, “Establishing Standards for Video Data Management,” at the annual meeting of the Society for Integrative and Comparative Biology in January 2017, with the primary goal of gathering community feedback on version 0.1 of the standards rubric. The workshop was on the last day of the meeting, so we had a few days before to distribute paper copies of the standards rubric, and to announce the workshop at the business meetings of the Divisions of Vertebrate Morphology, Comparative Biomechanics, and Animal Behavior. The workshop had 30 participants: 1 undergraduate, 10 graduate students, 6 postdocs, and 13 faculty members, and we received in-person, written, or e-mail feedback from four additional faculty members and a graduate student.

Feedback from the workshop was used to create version 0.5 of the standards rubric, and this version was distributed by e-mail to the workshop participants and other interested stakeholders. Version 0.5 generated some valuable comment threads, and these were used to create version 1.0, which was included in the peer-reviewed version of this article. Minor revisions were made in response to peer reviews, and version 2.0 is shown here as Table 1. We expect that the version here will be subject to future revisions, as required by changes in technology or community consensus on research needs.

The audience for these standards not only includes researchers and granting agencies, but also the developers and curators of video databases (e.g., Macaulay Library of wildlife media [macaulaylibrary.org], X-ray Motion Analysis Portal (xmaportal.org), and Zoological Motion Analysis Portal [zmaportal.org]) that may contribute to video data management and sharing efforts.

## Proposed standards for video data management

Based on the community-input process described above, we recommend a set of nine standards and three levels for good, better, and best practices in video data management for organismal biology research (Table 1). Level 0 is included to indicate an unacceptable, substandard level. Individual videos and projects can, and likely should, meet different levels for each standard, such as Level 2 for data storage, Level 1 for video file formats, Level 3 for metadata linkage, etc. Each research group and project is expected to have different needs for data management, and the standards and levels are designed to allow flexibility, while meeting a minimum of good (Level 1) data management practices. Data management levels may also vary through the life cycle of a project; our recommendations are generally targeted at the video data underlying research published in preprint or peer-reviewed form, though many of the recommendations are also good practice during data collection and analysis.

Below we describe the rationale and community consensus for each standard and level in Table 1.

### Data storage

Participants in the workshop had strong and somewhat conflicting opinions regarding standards for data storage. Some felt that local disk storage is never secure enough because people do not keep enough copies and all disk types fail. In this view, the minimum acceptable standard would be that video data must be placed in a dedicated data storage facility managed by IT professionals (university, commercial cloud, or research data repository). However, fees are often charged for such storage, failures occur even in these entities, and some early-career participants felt strongly that local disks are the least expensive solution and we should try to make Level 1 as accessible as possible to all researchers and students. The group settled on the current Level 1, allowing for either a local copy along with an archival copy in professionally managed storage or a doubly redundant local copy. Files stored on servers, managed or otherwise, should be subject to periodic fixity and data integrity checks; files on plain disks should be migrated to new media on a 3- to 5-year schedule. This minimum standard is based, in part, on a rubric for levels of data preservation proposed by the National Digital Stewardship Alliance, hosted by the Digital Library Federation (ndsa.org/documents/Levels_v1.pdf).

For the data storage standard, the distinction between Levels 2 and 3 arose after the workshop, in consultation with colleagues and a data preservation professional. Levels 1 and 2 implementations may include commercial cloud storage, such as Google Drive (google.com/drive) or Dropbox (dropbox.com), which generally must be paid for by individuals or their institutions (particularly for large data storage needs, as with video files). There is always a chance that companies can go out of business, or payments for the storage stop and the data become inaccessible. For this reason, Level 2 also includes the requirement to keep copies in two separate local storage systems. Level 2 can be fulfilled by noncommercial remote storage managed by IT professionals, such as is provided by some academic institutions, but in some cases this storage may also be fee-based and may not be part of a dedicated digital archive. In contrast, Level 3 depends on data repositories that have been created with a clear mission to preserve digital data. Most larger universities have digital data preservation resources, typically associated with the library or research computing units, and there are other data repositories such as Dryad (datadryad.org) and Open Science Framework (osf.io). Some of these may include fees to deposit data, and some not, but the stated mission of such repositories is to preserve data, so the expectation is that ongoing payments will not be required to keep data accessible. Level 3 does not require local copies to be kept, since the data repositories are assumed to be implementing high data-preservation standards, but it is wise to keep local copies for convenience if a repository is temporarily unavailable, such as for maintenance. A current problem with many data repositories is that they limit the maximum size of individual files, such as 5 GB for Open Science Framework, requiring that a single large video file be split into several subfiles for storage and reconstituted for use. High-resolution, high-bit-depth, high-speed and/or long video files are often larger than 5 GB, and will likely only get larger as technology continues to improve video quality.

### Video file formats

A standard for video file formats was not included in the workshop rubric version 0.1, but workshop participants suggested that the standards should include some guidance on this issue. We identified three goals associated with the selection of video file formats: (1) preserving original video data; (2) broad accessibility, now and for the future; and (3) ease of online viewing and downloading the (often very large) video files. These goals are somewhat incompatible, in that preserving original video data suggests that original file formats generated by the cameras should be kept, even if the format employs codecs or file types that are not widely accessible by common viewing software (in conflict with goal 2), or the files are very large or of a type not viewable online (in conflict with goal 3).

For video file formats, we propose prioritizing goal (1), preservation, by recommending that Level 1 be preservation of the original format in which the video was saved from the cameras, even if it is a proprietary camera manufacturer’s format or otherwise includes codecs or file types that are not widely accessible by common viewing software. Level 1 also acknowledges that converting video is time and disk-space consuming, and there is potential for data loss through decreasing bit depth, inadvertent overcompression, or inadvertent modification of pixel resolution, frame rate or time base when converting video formats. Level 2 adds storage of converted versions if the originals are proprietary or suspected to include file types or codecs that will lack accessibility or longevity. A folder of uncompressed TIFF images may currently be the most future-proof format, but we hesitate to recommend specific formats as video technology continues to change rapidly, and researchers and database creators should have the flexibility to select file formats. Level 3 adds compressed or converted versions for greater accessibility online, particularly if the videos are accessible through an interface that allows viewing the videos online. As noted in a footnote to Table 1, Levels 1–3 are identical if original video files are already in a widely accessible format and small enough for easy viewing and accessibility online.

The timeline for evaluating when to migrate data from one file format to another as new and more sustainable formats become available will vary. Selection and appraisal are key parts of a digital preservation strategy (Harvey 2008). Every 3–5 years, researchers should appraise their data collections to determine which files are stored on media or in formats that are at the highest risk of succumbing to degradation and/or technological obsolescence (no longer widely supported or replaced by newer versions), and also determine which collections, or individual files within them, should be prioritized for migration and long-term preservation. There may come a point (after publisher, granting agency, and institution mandated research data and records retention periods have expired) when researchers may choose to “weed” or no longer actively manage some of their locally stored data. These preservation decisions may be based on several factors: the identification of data determined to no longer have significant scientific or historical value; the identification of media and/or files containing data that have changed or degraded to the point of being unrenderable (e.g., media or bit rot); the determination that the cost for locally storing, backing up, and mirroring the files is no longer economically sustainable; or the identification of data that are no longer unique, that is, a copy of the data, or a similar or higher quality dataset, exists in a repository managed by IT professionals (Harvey 2008; Whyte and Wilson 2010).

### Metadata linkage

Standard 3, focusing on metadata linkage, addresses the establishment and maintenance of connections between video files and their metadata (the actual metadata to be preserved are described in standards 5–9). Participants in the workshop expressed concern about the issue of keeping metadata properly associated with the video files, so this standard was changed from “Metadata Access” to “Metadata Linkage” to emphasize the linkage issue. For sharing and access, metadata should be stored in digital files, rather than just in paper lab notebooks. The digital files should be in a widely accessible format. Non-proprietary formats are preferred, such as plain or Rich Text documents and comma-separated values for spreadsheets, but some widely used proprietary formats, such as Microsoft Office file types, are also acceptable, particularly if open source editors are available.

Typically there should be one metadata file for each video file, or file set in the case of calibrated, multi-camera recordings. These digital metadata files need to be connected to the individual video files, and this linkage can be maintained in one or more of three ways: using similar file names with a carefully designed file-naming convention that clearly marks the video file and its metadata file as connected; bundling them together at the file level in an uncompressed archive; or placing the metadata into the video file itself, as in the extensive headers permitted by some video file formats (although such linkage can make the video files less accessible to some users). Level 1 of the metadata linkage standard specifies use of file names OR bundling, and Level 2 specifies use of file names AND bundling, based on the value of having the file names to connect the files even after they are unbundled. Level 2 also offers placement of metadata within the video files as an alternative.

Level 3 of the metadata linkage standard specifies that the metadata (including video file name) be encoded in a machine-readable format, such as XML, and placed within the video file or bundled with it. Making the metadata machine readable may facilitate future automated analysis of the data, and particularly would facilitate importation of metadata and video data into future databases. Databases form the back end for web interfaces that allow video display, searching and analysis, and automated routines can populate the database metadata fields from the machine-readable metadata. Examples of such databases with web interfaces are the XMAPortal (xmaportal.org) and ZMAPortal (zmaportal.org), hosted at Brown University and managed by one of the authors of this piece (E.L.B.), and Macaulay Library of wildlife media (macaulaylibrary.org).

In addition to the formats of video files and their linkage to metadata, video and metadata “file names” can be important for accessibility and ease of machine-reading and use. Encoding the minimum essential metadata in file names can also be a way to ensure that metadata remain linked to the file (see an example naming scheme, below). We do not include file-naming recommendations in the standards (Table 1), with the understanding that individual research groups and projects may have specific needs for file-naming conventions, including existing code bases that depend on a specific naming scheme. But we offer these recommendations here as guidance. Standard recommendations include: only one period in file names, with the period before the extension; no spaces or special symbols in the file names; use the expected maximum number of digits for numbers, for example, 001, 010, and 100 for a sequence of 1-100; and use the international standard ISO 8601 for date format (YYYYMMDD) (iso.org/iso-8601-date-and-time-format.html). In addition, we highly recommend encoding the minimum essential metadata in the filename itself, even if that means creating long file names such as Perameles_gunnii_03_postImplant_20170423_r12_300fps_cam2.mp4. This naming scheme is presented here just as an example, and may be generalized as Genus_species_individual_experimental treatment (or other attribute of the recording)_date_run number (or trial number)_frame rate_camera number. Such file names are one more strategy to ensure that essential metadata remain linked to the video file, but do not eliminate the need for separate and more complete metadata files.

### Video data and metadata access

Standard 4, video data and metadata access, is concerned with whether the videos and metadata are available and discoverable online. Level 1 requires that data be available online and potentially accessible by other users. This lowest acceptable level is inconsistent with the lowest acceptable level for Standard 1 (data storage), which permits local storage only, but as mentioned above, individual research groups and projects can address each standard individually. Participants in the workshop discussed the issue of internal consistency without reaching consensus. We feel that it would, for example, be worthwhile to meet the lowest data storage standard, even if some of the other minimum standards are not met. Level 2 of this standard requires depositing data in a repository with a stated mission of providing public access to data, and Level 3 adds the stipulation that metadata should be discoverable. For data to be discoverable, they should not be exclusively bundled into archives, but should also be able to be viewed without having to download large files. Best for sharing and video data discovery would be the placement of metadata and videos in a database designed to make them discoverable from outside the site, and with a web interface to make the metadata searchable and viewable and the videos viewable from within the interface (e.g., XMA/ZMAPortal, and Macaulay Library of wildlife media [macaulaylibrary.org]).

### Contact information and acceptable use

Standards 5–9 all are concerned with specific metadata to be recorded and kept with the video files. Meeting Level 1 for all of them would meet a “minimum information standard” (e.g., Brazma et al. 2001) for the minimum metadata required to make most videos reusable for organismal biology research. For contact information and acceptable use (Standard 5), minimum (Level 1) would be contact and rights information (i.e., identity of the copyright owner, e.g., the researcher, the journal or publisher, an institution) and a statement of acceptable reuse, and best (Level 3) would be the ORCID ID (orcid.org) of a contact person, rights statement, assignment of an internationally-recognized content license such as Creative Commons (creativecommons.org), and a unique and persistent identifier, such as a digital object identifier (DOI). The DOI could be assigned to a single video, but more likely would be assigned to a collection of videos belonging to a specific study, project, or publication. Standard 5 is critical for researchers who would like to reuse others’ video data. Data consumers need to know the identity of the rightsholders in case they need to seek any additional permissions not outlined in their terms of use, as well as have the identity of the data creators and the DOI of the dataset in order to cite and provide them with attribution (Wilkinson et al. 2016). Lastly, knowing the identities of data creators as well as the provenance of publicly available video datasets assists data consumers in their selection and appraisal of these data.

### Camera settings

Minimum metadata for camera settings (Standard 6) is frame rate, since the vast majority of video studies in organismal biology have a time component, such as measuring velocity, frequency, or rate of occurrence of some behavior. Some studies can do without a time base, which opens up YouTube and other vast sources of online videos of organisms, and pets in particular, for scientific use. Spatial calibration data, Level 2, are also required for many studies, and suggestions for other potentially useful camera and recording metadata are included in Level 3. In some cases, metadata automatically recorded from cameras (often in EXIF (EXchangeable Image File) format) may contain useful metadata that could be harvested to meet some of these metadata standards.

### Organism(s)

The minimum (Level 1) metadata for organism(s) (Standard 7) are genus and species, with Levels 2 and 3 suggesting other potentially useful organismal metadata.

### Recording conditions

The minimum metadata required for recording conditions (Standard 8) are less clear than those for organism(s), and may depend on the specific study. International standards, such as ISO 8601 for dates (YYYYMMDD), are available for some of these metadata, and should be used whenever possible. Date is valuable for identifying videos as belonging to a specific lab experiment or field project, although the title of the project should be included in other metadata fields (see “Subject Matter/Topic” below) as well. Location is required for some studies, but quite irrelevant for others. However, even though the minimum metadata for the recording conditions standard are hard to define, it is worth including this standard for all the suggestions made for Level 3 that might be valuable for specific projects. Again, EXIF metadata from cameras may be useful, such as GPS (Global Positioning System) coordinates.

### Subject matter/topic

The final standard discussed in the workshop concerns the subject matter/topic of videos. This standard is mostly aimed at making video data discoverable, but some of the metadata may also enhance the reusability of the data. Important elements to include would be an abstract with any related publication citations or other relevant and related citations, such as for locations and identifiers of software and data analysis scripts, and funding information and acknowledgments. Best practice (Level 3) would be to select keywords from an internationally recognized subject list, such as the Encyclopedia of Life’s TraitBank (eol.org/data_glossary) or National Library of Medicine’s Medical Subject Headings (MeSH) (nlm.nih.gov/mesh/MeSHonDemand.html). However, for such lists to be most valuable, the field should develop consensus on which list(s) to use, a task that is beyond the scope of the current consensus-building project (but see the section, “Next steps,” below).

## Example applications of the video data management rubric

In the following narratives, we provide descriptions of how the rubric can be applied to individual studies. These real-world studies provide examples of a range of levels that were met through the course of research conducted under various circumstances, and highlight how use of the rubric can call attention to specific issues that can be addressed in efforts to use best practices. Details of how each study meets, or does not meet, the recommended levels of each standard are provided in Table 2. All of these studies were conducted and published before the creation of the rubric. Nonetheless, applying the rubric to them post hoc is informative.

**Table 2:** Examples of the video data management rubric applied to published works

Standards	Example 1	Example 2	Example 3
Example source	van Leeuwen et al. (2015)	Mayerl et al. (2016)	Evangelista et al. (2017)
	Dryad Data DOI: http://dx.doi.org/10.5061/dryad.r503m.	Data archived at xmaportal.org: unique Study ID Brown40.	Dryad Data DOI: http://dx.doi.org/10.5061/dryad.p68f8.
(1) Data storage	Level 2: Original, archival files on IT-managed storage server (triple backup) at the co-PI’s institution, on DVDs and external hard drives at PI’s and co-PI’s institutions, and cropped images (to reduce file size) on Dryad data repository (not archival).	Level 3: XMAPortal (data repository), storage on Google drive, two external hard drive copies at co-PI's institutions.	Level 2: Maintained in IT-managed storage at UNC, a network accessible lab working copy, and an off-network lab backup; incomplete video set also on Dryad data repository with the kinematics.
(2) Video file data	Level 2: Uncompressed original format (tiff images) on Dryad, description of image processing on Dryad (cropping); original uncompressed format (tiffs) on storage server.	Level 3: Original cine files stored in the XMAPortal; XMAPortal interface allows video viewing online, as well as download of videos converted to user-selected formats.	Level 2: Original camera output, in this case the widely accessible .MOV container with h.264 compression.
(3) Metadata linkage	Level 1: Metadata of all videos (video format, description of video sequences) available on Dryad as text files (dat and txt format), metadata not bundled with video file, but in a separate metadata folder.	Level 3: Metadata contained within original cine files; metadata downloadable from XMAPortal with same file names as associated videos and bundled into zip archives.	Level 2: Metadata available as plain text on Dryad and in the .MOV container headers; including frame rate, resolution, UTC recording time and GPS location.
(4) Video data and metadata access	Level 2: Videos and metadata available on Dryad.	Level 3: Videos and metadata available through XMAPortal; metadata searchable and videos and metadata viewable within the Portal interface.	Level 1: Metadata and an incomplete video set on Dryad; complete video set in a shareable and internet-accessible location.
(5) Contact information and acceptable use	Level 2: Contact details and statement provided about how to cite the data on Dryad with CC0 licensing through Dryad and DOI through Dryad.	Level 3: Names, contact e-mails, ORCID IDs and CC BY 4.0 licensing provided in study metadata through XMAPortal; study assigned unique identifier in the XMAPortal.	Level 3: ORCID IDs associated with data in Dryad with Dryad CC0 licensing and DOI through Dryad.
(6) Camera settings	Level 1: Metadata documents on Dryad give frame rate and calibration factor (meters/pixel) given in separate file, one file per video recording.	Level 2: Frame rate, spatial calibration, and camera ID available, with some additional information (X-ray settings, shutter speed).	Level 1: Single-camera settings available in file headers; multi-camera calibration and lens distortion data in an internet-accessible location with complete video data.
(7) Organism(s)	Level 2: Species and age provided; additional data (size) provided in publication but not on Dryad.	Level 2: Genus, species, size, and individual ID provided.	Level 1: Genus, species provided on Dryad.
(8) Recording conditions	Level 1: Date of recording provided; location and context provided only in associated publication, not in the metadata document on Dryad.	Level 2: Date and location of recording provided, as well as environment; people who recorded video available through high level of database menu.	Level 2: Date and location encoded in camera metadata; these plus the environmental context are also provided in the publication.
(9) Subject matter/topic	Level 1: Abstract on Dryad.	Level 1: Association of video with project identified through XMAPortal; text description of behavior provided with menu listing of each video.	Level 2: Subject matter, behavior, and original recording purpose described in Dryad metadata.

### Example 1

Example 1 is based on video data used in a publication (van Leeuwen et al. 2015) on the body dynamics of swimming fish. The study calculated body dynamics from digitized midlines of zebrafish larvae during cyclic swimming to explore how swimming dynamics change with age and body size. This study used both old video data from a previous publication (Müller and van Leeuwen 2004) and new video data. The old video data were recorded in 2002 and were archived on DVDs (Digital Versatile Disc) (duplicate) and on external hard drives (duplicate) in two different locations. Processed data (digitized midlines of the fish) were archived on external hard drives (more than 10 copies) in two different locations. Until 2010, the old video data had not been backed up on a storage server or in cloud storage. Both authors of the 2004 publication lost some copies of the video data during moves to new institutions or to a new building in 2007 and 2008. They were able to find at least one copy of all but one of the videos that formed the basis of the 2004 publication. Since this incident, both investigators have archived their video data on storage servers. This episode illuminates the importance of creating multiple backups, including storage servers or cloud storage. It also illustrates a common tendency for researchers to store processed data (in this case: the digitized midlines of the fish body) rather than original videos, because processed data require orders of magnitude less memory.

While this example scores well enough with regard to data storage (rubric standard 1), it scores lower in several standards for metadata archiving and sharing (Table 2, Example 1). The authors have archived metadata (rubric standards 6–9) for this study in paper lab notebooks and electronic inventories, but this information is neither easily shareable nor particularly well archived (paper lab notebooks exist only as a single copy plus as a scanned digital version). Sharing and archiving might be facilitated within a research team by using digital lab notebooks. However, access to metadata beyond the research team would have been better if the authors had had access to and had used the proposed rubric when assembling the metadata file archived on Dryad.

### Example 2

Example 2 focuses on a recent study that used X-ray videos to compare the magnitude of pelvic movements in representative species from two lineages of turtles during swimming and terrestrial walking (Mayerl et al. 2016). In one lineage (the pleurodires) the pelvis is fused to the shell, so that no movement was expected. The pelvis is not fused to the shell in the other lineage (the cryptodires), but it was unclear whether soft tissue attachments, or the construction of the shell itself, might restrict the potential for pelvic movements to contribute to hind limb stride length. Radio-opaque markers were implanted into five turtles before filming, and videos were coordinated with computed tomography (CT) scans to allow the use of X-ray reconstruction of moving morphology (XROMM) to measure kinematics (Brainerd et al. 2010). All videos used in this study were newly collected.

In the course of data collection, videos (2–6 GB per video) and associated metadata were deposited into the XMAPortal (xmaportal.org), a searchable online database developed as part of an NSF Research Coordination Networks project, and maintained through the home institution (Brown University) of one of the study co-authors (E.L.B.). This repository was specifically designed to maximize the preservation and accessibility of large video files and metadata, and scores highly with respect to the standards of the proposed rubric (Table 2, Example 2). Storage through the XMAPortal promotes data archiving, viewability, and sharing through download options of studies made publicly available (Standards 1, 2, and 4). The XMAPortal also enables the linkage of metadata to video files, both online in the database interface and for download with the metadata bundled into zip archives with the associated videos (Standard 3). Fields are also available within the portal for the entry of contact and use information (Standard 5) and a wide variety of metadata on both camera settings and study subjects (Standards 6–9). However, Table 2 shows that, even with the considerable aid imparted by a guiding framework like the XMAPortal, it is still up to investigators to ensure the entry of a full range of metadata for association with videos. Use of the proposed rubric can help guide such entries and maximize the potential for future use of video data.

### Example 3

Example 3 is from a recent study of Chimney Swift flock behavior that used multi-camera videography to record the trajectories of ∼1800 birds during their flock formation, circling, and chimney landing behavior (Evangelista et al. 2017). The cameras used were 3 Canon EOS 6D running in movie mode, which produces a lightly compressed QuickTime (i.e., MOV) format video in 24-bit color with an Advanced Audio Coding (AAC) audio track. As such, the original video file format met the Level 2 standard (Table 2, Example 3). During data analysis and manuscript preparation, the data were stored on a lab server housed in a university IT facility with nightly backup to a second server in a different building. Following publication the video data and associated analysis files were also copied to a departmentally managed storage system with online and offline backup [data storage, Level 2]. Metadata were stored in a plain text file in the folder with the video and subsequent analysis files; camera-associated metadata were included in the file headers and field notes recorded in the audio track [metadata linkage, Level 2]. The flock trajectory kinematics files (2 GB in total), metadata and the video files from one of the three cameras (12 GB) were deposited in the Dryad archive [video data and metadata access, Level 1] and made publically available with a CC0 license as required by the Dryad archive [contact information and acceptable use, Level 3]. Metadata details such as camera settings, recording conditions, organisms and subject matter generally meet Level 1 or 2 standards. In general, data sharing efforts for this project focused on making the resulting kinematics rather than the raw video available. The video data capture no detail of the animals themselves; swifts are small black dots a few pixels wide under the recording settings chosen for the project, and essentially produce only the position time-series more compactly shared as the 3D kinematics. Nevertheless, video may provide important contextual information for flock behavior so one of the camera views was added to the Dryad archive. Preservation of the full video dataset for further analysis or sharing with users who wish to replicate the initial 3D reconstruction and time-series construction steps is also desirable, thus the data storage standard used here is higher than the video data sharing standard. Finally, on the whole Dryad is currently a better repository for sharing and discovering numeric data than video data; large videos such as those generated here must be split into 1 GB chunks and reassembled after download, a command-line operation in most cases.

## Next steps

Given the potential for rapid changes in video technology and data science, it is clear that any community-established standards for video data management and preservation should undergo frequent review and revision to ensure that they remain a useful framework. As in this initial effort, there is a strong motivation for such review and revision to come from the community of organismal biology researchers (Yarmey and Baker 2013). Approaching standards as a “living document,” potentially with a web-based interface for receiving input, could facilitate this process.

For metadata organization, standardization, and linkage to video files, a next step for the organismal biology research community would be to develop an XML (or similar machine-readable) template for storing video metadata. An XML template would standardize metadata fields, could be disseminated widely, and facilitate the creation of open-source tools for generating the metadata files. These open-source tools would be digital lab or field notebook tools that prompt users to enter specific metadata and also allow flexibility for entering free text or numbers when needed. The XML template, in turn, should rely on existing standards and ontologies/controlled vocabularies or on a new controlled vocabulary for video metadata created by the organismal biology community.

The metadata field names and controlled vocabularies developed or adapted for the XML template could then be used by database developers to specify the database fields in online video data repositories for organismal biology. Two such video data repositories for organismal biology have already been developed, the X-ray Motion Analysis Portal (xmaportal.org) and the Zoological Motion Analysis Portal (zmaportal.org). These repositories were developed with NSF funding, are hosted by Brown University, and are available for researchers to use now (see Example 2 above). The metadata fields specified in the XMA/ZMAPortal benefitted from one workshop held at Brown with 25 invited researchers and students. But further input from the research community would refine and standardize metadata field names and controlled vocabularies for the XML template that could then be incorporated into XMA/ZMAPortal or other specialized video data repositories.

In addition to being video data repositories with a commitment to data preservation, the XMA/ZMAPortal are also “data management tools” designed to be used before, during, and after data collection to increase the efficiency of working with large (>2 GB) video files. The XMA/ZMAPortals offer online software tools for experimental design, for recording metadata in digital lab notebooks during data collection, and for video review and annotation with an online Multi-Cam Viewer tool. The Multi-Cam Viewer displays downsampled videos from up to eight synchronized cameras simultaneously (downsampled for rapid online viewing), with the ability to call up individual high-resolution frames. The XMA/ZMAPortals store all original videos, in original camera video formats, and also provide versions converted on the fly to a file format requested by the user. The code that does the conversion can be updated in the future and new formats added, as needed. The XMA/ZMAPortals also support automated data export to open-source XMALab software for motion analysis, and then re-importing the tracked motion data to XMA/ZMAPortal with metadata about the analysis (Knörlein et al. 2016).

The XMA/ZMAPortals are designed to export and import metadata in XML files for interoperability among databases and data analysis tools. Users can create their own specialized programs for entering metadata into XML files during experiments (i.e., customized digital lab notebooks), and then create pipelines to upload videos and populate the metadata fields in the XMA/ZMAPortal. Users can download video data bundled with XML files of metadata that can then feed into specialized motion analysis pipelines, with essential metadata, such as frame rate, read automatically from the XML files.

The curation standards for video data and reporting template for video metadata eventually adopted by the organismal biology research community could be mapped to resources like BioSharing.org. Granting agencies could use them to align their expectations for researchers' management and sharing of video data with community practices. Lastly, scholarly journals in the field could use these standards to create policies for authors to consult before preparing and depositing their video files in a data repository, and before citing their own or others' video data in their publications.

This next step of developing an XML template with standardized fields and vocabulary for storing video metadata should be undertaken by the organismal biology research community as soon as possible. We offer the process we developed here—including pre-workshop communication with stakeholders, workshops at meetings of large scientific societies to gather more opinions and build consensus, post-workshop communication with participants for further refinement, and finally long-term input into standards as living documents—as an example of community-consensus building for data and metadata standards.
